# Serum IL-10 Predicts Worse Outcome in Cancer Patients: A Meta-Analysis

**DOI:** 10.1371/journal.pone.0139598

**Published:** 2015-10-06

**Authors:** Shuai Zhao, Dang Wu, Pin Wu, Zhen Wang, Jian Huang

**Affiliations:** 1 Cancer Institute (Key Laboratory of Cancer Prevention & Intervention, National Ministry of Education; Provincial Key Laboratory of Molecular Biology in Medical Sciences), Zhejiang University, Hangzhou, China; 2 Department of Oncology, Second Affiliated Hospital, Zhejiang University School of Medicine, Zhejiang University, Hangzhou, China; 3 Department of Thoracic Surgery, Second Affiliated Hospital, Zhejiang University School of Medicine, Zhejiang University, Hangzhou, China; Shanghai Jiao Tong University School of Medicine, CHINA

## Abstract

**Background:**

IL–10 is an important immunosuppressive cytokine which is frequently elevated in tumor microenvironment. Some studies have reported that overexpression of serous IL–10 is correlated with worse outcome in patients with malignant tumor. Here, we conducted a meta-analysis to assess the prognostic impact of serous IL–10 expression in cancer patients.

**Methods:**

We searched PubMed and EBSCO for studies in evaluating the association of IL–10 expression—in serum and clinical outcome in cancer patients. Overall survival (OS) was the primary prognostic indicator and disease-free survival (DFS) was the secondary indicator. Extracted data were computed into odds ratios (ORs) and 95% confidence interval (CI) or a P value for survival at 1, 3 and 5 years. Pooled data were weighted using the Mantel–Haenszel Fixed-effect model. All statistical tests were two-sided.

**Results:**

A total of 1788 patients with cancer from 21 published studies were incorporated into this meta-analysis. High level of serum IL–10 was significantly associated with worse OS at 1-year (OR = 3.70, 95% CI = 2.81 to 4.87, P < 0.00001), 3-year (OR = 3.33, 95% CI = 2.53 to 4.39, P < 0.0001) and 5-year (OR = 2.80, 95% CI = 1.90 to 4.10, P < 0.0001) of cancer. Subgroup analysis showed that the correlation between serous IL–10 expression and outcome of patients with solid tumors and hematological malignancies are consistent. The association of IL–10 with worse DFS at 1-year (OR = 3.34, 95% CI = 1.40 to 7.94, P = 0.006) and 2-year (OR = 3.91, 95% CI = 1.79 to 8.53, P = 0.0006) was also identified.

**Conclusions:**

High expression of serous IL–10 leads to an adverse survival in most types of cancer. IL–10 is a valuable biomarker for prognostic prediction and targeting IL–10 treatment options for both solid tumors and hematological malignancies.

## Introduction

Chronic inflammation is closely linked to cancer [1–4]. Cancer-related inflammation promotes the development and progression of tumor by different mechanisms, specifically by subverting immune response, inducing gene mutations, stimulating angiogenesis and cell proliferation and inhibiting apoptosis [5, 6]. Multifunctional cytokines are a significant mediator in the development of malignant tumor by participating in bidirectional regulation of inflammatory responses [7, 8]. A large number of studies demonstrated that cytokines could facilitate carcinogenesis by both provoking inflammation [9–11] and eliciting immunosuppression [12–14].

Interleukin 10 (IL–10) is an immunoregulatory cytokine mainly produced by regulatory T cells and helper T cells [15, 16]. The primarily function of IL–10 is initially considered as an effective anti-inflammatory cytokine, which functions through suppressing macrophage/T cell cytokine expression and inhibiting their antigen-presenting capacity by activating STAT3 signal pathway [17–19]. Accumulating evidence showed that IL–10 played a pleiotropic role in both immune stimulation and suppression in tumor inflammatory microenvironment [20–23]. As an inflammatory modulatory cytokine, IL–10 was reported to exert both anti-tumor and pro-tumor function [19, 24, 25]. In previous studies, high level of IL–10 was reported to correlate with poor survival of some cancer patients [26–29], while some other studies provided opposite results [30–32]. Both human recombined IL–10 and IL–10 antagonist have been launched for cancer therapy [33–36]. Nowadays, deep insight into the controversial functions of IL–10 in cancer is urgently needed. Moreover, the potential of IL–10 as an effective biomarker in prognostic prediction and targeted therapy is necessary to be explored.

Here, we performed this meta-analysis to test OS and DFS as outcomes in cancer patients with known serum IL–10 levels. The purpose of this study was to quantitatively summarize the association between serum IL–10 overexpression and clinical outcomes in cancer patients, and thereby shed more light on the clinical value of IL–10 as a prognostic biomarker and therapeutic target for both solid and hematological malignances.

## Methods

### Search and Selection of Studies

We searched PubMed and EBSCO for studies evaluating the expression of IL–10 in serum and survival in cancer patients from 1993 to December 2012. The research term was ("Neoplasms"[Mesh]) AND ("Interleukin–10"[Mesh]). Results were restricted to serum IL–10 detection in human cancer. A total of 2023 and 3091 entries were identified in PubMed and EBSCO respectively. Inclusion criteria were the measurement of IL–10 by Enzyme-Linked Immunosorbent Assay (ELISA), available data of overall survival (OS) or disease free survival (DFS) for at least 1 year, and publication in English. Exclusion criteria included studies evaluating gene expression of IL–10, detecting IL–10 in tissues, and studies on animals or in lab. The association between IL–10 and survival was the primary consideration for study selection. The most complete study was chosen when a clinical trial had more than one publication. All the studies selected were assessed by the Newcastle-Ottawa Scale (NOS).

### Endpoints of Interest

OS at 1, 3 and 5 years was recorded as the primary outcome of interest, and DFS at 1 and 2 years was recorded as the secondary clinical outcomes. Cut-offs of IL–10 expression level defined by individual studies classified cancer patients into high- and low- groups

### Data Collection

Data were independently extracted by two authors (Shuai Zhao and Dang Wu) using standardized data abstraction forms. The following details were collected from individual studies: types of tumor, patient numbers, time of follow up, technique for IL–10 detection, and cut-off to determine IL–10 positivity. In all cases, survival data were extracted from tables or Kaplan–Meier curves for both IL–10 negative (control group) and IL–10 positive (experimental group) patients.

### Data Synthesis

All inclusion study data were pooled in this meta-analysis initially. Odds Ratio (OR) and its 95% confidence interval (CI) were derived to express the relative frequency of survival at 1, 3 and 5 years between the negative and positive groups. Two subgroups were generated for patients of hematological malignancies and solid tumors.

### Statistical Analysis

Extracted data were combined into a meta-analysis using RevMan 5.3 analysis software (Cochrane Collaboration, Copenhagen, Denmark). Pooled estimates of ORs were computed using the Mantel–Haenszel fixed-effect model. Cochran’s Q and I^2^ statistics were used to assess statistical heterogeneity. Differences between the subgroups were assessed using methods as described previously by Deeks et al [37]. Batch Effects was assessed between overall survival and batch using ComBat. Sensitivity analyses were performed for batch correction and different cut-offs in defining IL–10 expression to assess the robustness. All statistical tests were two-sided, and P value less than 0.05 are considered statistically significant.

## Results

### Search results

**Fig 1:**
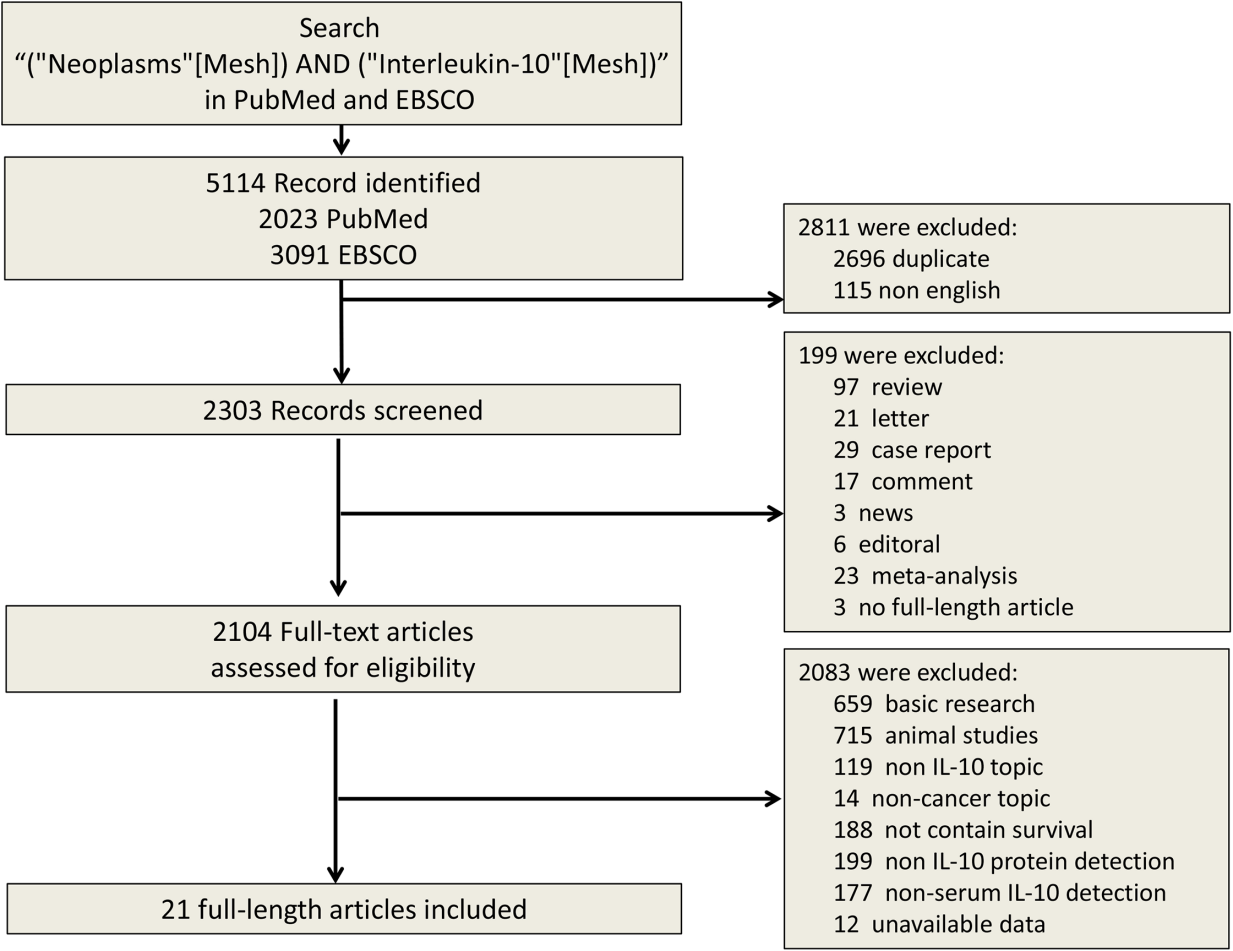
Selection of studies included in the analysis. IL–10 = interleukin–10.

The search results have been shown in Fig 1. The primary literature research retrieved 5114 records. After screening the title of citations, 3010 records were excluded because of the non-relevance with the theme and duplicated literatures. Next, 2083 citations were excluded after screening abstracts of the records. Then we read carefully the full text of the left citations and at last 21 studies were included.

### Description of studies

21 studies including 1788 patients were identified for the assessment of IL–10 expression [38–58]. All the studies were evaluated by the Newcastle-Ottawa Scale (NOS), and were in accordance with the inclusion criteria and suitable for data consolidation. Table 1 and Table 2 show characteristics of included studies for OS and DFS respectively. Seven studies evaluated lymphoma, two evaluated lymphocytic leukemia, five evaluated gastrointestinal cancer, three evaluated hepatocellular carcinoma, and one each evaluated head and neck cancer, pancreatic cancer, melanoma, and lung cancer. Eighteen studies reported data for 1-year OS, 3-year OS and 5-year OS and three studies reported data for 1-year DFS and 2-year DFS.

**Table 1:** Characteristics of the included studies for OS analysis.

References	Type of cancer	Patient NO.	Age(range)	Male/female	stage	cut-off	Follow up months (range)	IL–10(-/+) NO.	1-y OS(-/+)%	3-y OS(-/+)%	5-y OS(-/+)%	Quality Score (NOS)
Alhamarneh, O., et al.(2011)[38]	head and neck squamous cell carcinoma	107	64(26–90)	85/22	I-IV	0.2 pg/ml	15(1–36)	66/41	90/78	74.4/52.8	NR	8
Blay, J. Y., et al.(1993) [39]	Non-Hodgkin’s Lymphoma	70	46 (3–89)	NR	I-IV	100 pg/mL	NR	32/38	81.4/67.9	78/53.6	78/49.3	8
Chan, S. L., et al. (2012) [40]	hepatocellular carcinoma	222	59.9±12.3	198/24	I-IV	1.00 pg/mL	31(0–48)	146/76	57.7/25.6	25.6/7	NR	8
Cortes, J. E., et al. (1995) [41]	Diffuse Large Cell Lymphoma	52	56(24–78)	32/20	I-IV	8 pg/ml	26(12–44)	26/26	75.5/73.6	43.7/61.4	NR	7
De Vita, F., et al. (1999) [42]	Gastrointestinal Malignancies	58	<60:50%. >60:50%	39/19	III-IV	18 pg/mL	12.9±6.4	29/29	92.4/32.1	NR	NR	7
De Vita, F., et al. (2000) [43]	Non-small Cell Lung Cancer	60	<60:46.7%. >60:53.3%	49/11	III-IV	19.6 pg/mL	12.8±7.8	21/39	74.3/9.6	5.1/0	NR	8
Ebrahimi, B., et al. (2004) [44]	Pancreatic Carcinoma	50	65(43–79)	30/20	NR	9.8 pg/ml	10(0–22)	9/41	44/0	NR	NR	7
Evans, C., et al. (2006) [45]	colorectal cancer	33	76.5	22/11	NR	NR	89	17/16	59/53.1	41.2/23.6	37.5/17.6	7
Fayad, L., et al. (2001) [46]	chronic lymphocytic leukemia	159	60(21–82)	NR	I-IV	10 pg/ml	30(1–40)	54/105	96.4/78.3	85/45	NR	8
Green, V. L., et al. (2012) [47]	head and neck squamous cell carcinoma	106	64(26–90)	84/22	I-IV	0.2 pg/ml	33(1–67)	64/42	85.3/73.3	74.4/54.6	59.1/54.6	8
Hattori, E., et al. (2003) [48]	hepatocellular carcinoma	74	65(41–88)	54/20	I-IV	10 pg/ml	NR	35/39	45/16.8	15.5/0	NR	8
Lech-Maranda, E., et al. (2010) [49]	Diffuse Large B-Cell Lymphoma	106	<60:46.2%. >60:53.8%	50/56	I-IV	5 pg/ml	NR	24/82	NR	70.7/36	NR	8
Lech-Maranda, E., et al. (2012) [50]	chronic lymphocytic leukemia	160	<60:32.5%. >60:67.5%	86/74	I-IV	17.8 pg/ml	48(1.2–200)	82/78	NR	NR	92.1/80.52	8
Nacinovic, A., et al. (2008) [51]	diffuse large B-cell lymphoma	46	58 (17–82)	25/21	I-IV	20.2 pg/ml	50(1–69)	16/30	85.8/74.9	75/31	71/25	7
Nemunaitis, J., et al. (2001) [52]	Melanoma	41	NR	NR	advance	10 pg/ml	NR	18/23	60.5/28.1	27.3/11.4	NR	7
Szaflarska, A., et al. (2009) [53]	Gastric Cancer	136	61.5±11.9	84/52	I-IV	10 pg/ml	82.6	49/87	71.6/52.9	55.4/26.2	NR	7
Vassilakopoulos, T. P., et al. (2001) [54]	Hodgkin’s lymphoma	122	31.5(15–76)	74/48	I-IV	10 pg/mL	27(6–136)	55/67	93.4/79.7	88.3/75	85.4/66.5	8
Viviani, S., et al. (2000) [55]	Hodgkin's disease	73	27(17–61)	44/29	I-IV	6 pg/ml	7.3y(1-8y)	33/40	100/87.8	95.4/82	95.4/79	8

**Table 2:** Characteristics of the included studies for DFS analysis.

References	Type of cancer	Patient NO.	Age(range)	Male/female	stage	cut-off	Follow up months (range)	IL–10(-/+) NO.	1-y OS(-/+)%	2-y OS(-/+)%	Quality Score (NOS)
Chau, G. Y., et al. (2000) [56]	Hepatocellular Carcinoma	67	63.4±1.5	60/7	NR	IL–10 >12 pg/mL	NR	21/46	66.3/46.6	62/31	8
Galizia, G., Orditura,M., et al. (2002) [57]	Colon Cancer	30	65.4±10.5(30–83)	NR	NR	IL–10 >15 pg/mL	22.2±6.6(5.2–26.1)	15/15	93.3/86.2	93.2/60	7
Galizia, G., Lieto,E., et al. (2002) [58]	Colon Cancer	50	65.4±10.5 (37–83)	34/16	NR	IL–10 >14 pg/mL	15.5±6.7 (0.3–26.1)	25/25	100/75.5	83.4/67.3	7

### Evaluation and Expression of IL–10

Serum level of IL–10 in samples was tested by ELISA in companies according to the manufacturer’s instructions. The cut-off for high level depended on the serum concentration of IL–10. No significant difference of IL–10 level was observed among different types of cancer.

### Association of IL–10 with Survival

**Fig 2:**
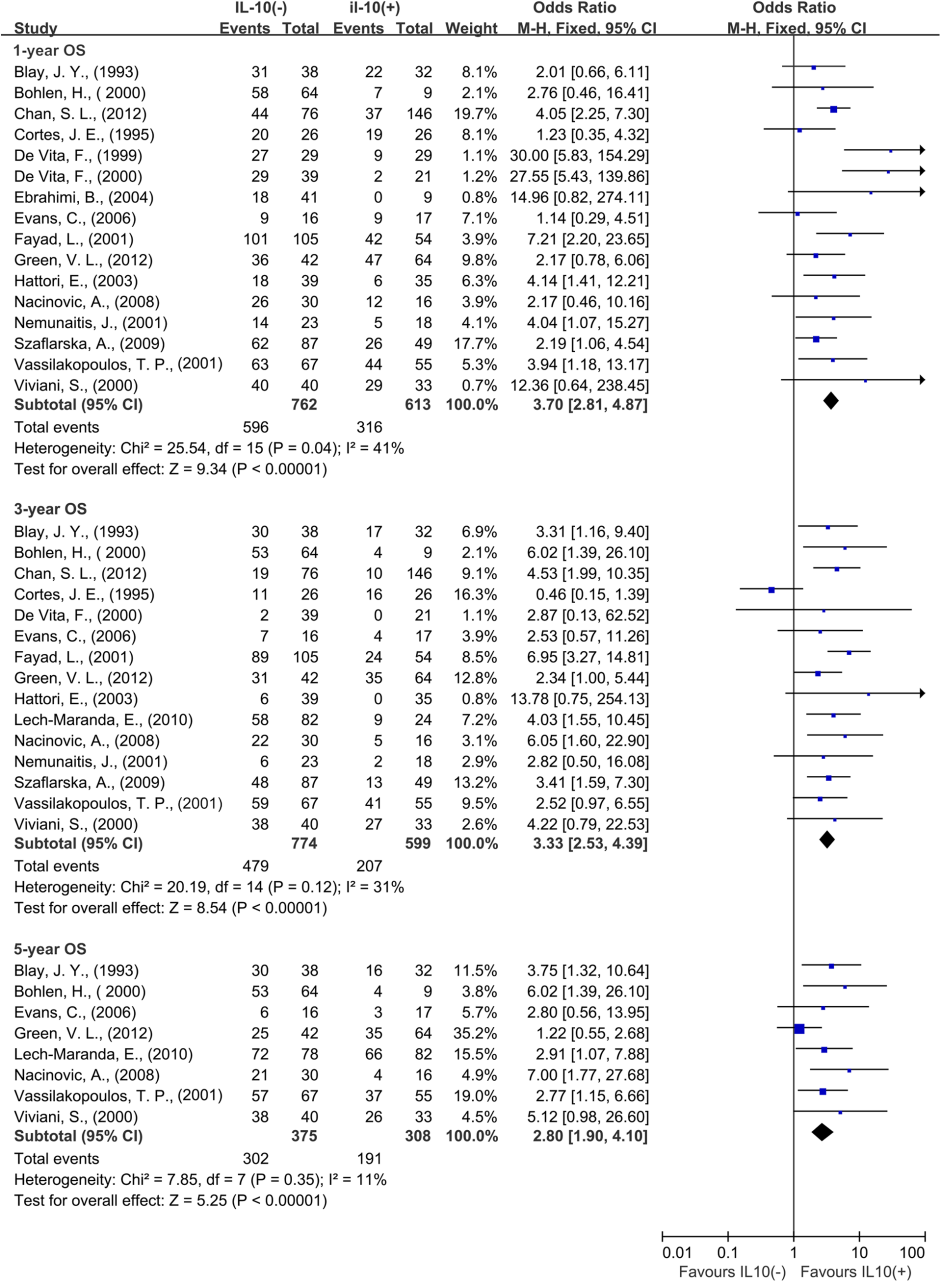
Forest plots describing odds ratios of the association between serous interleukin–10 (IL–10) expression and overall survival (OS) at 1, 3 and 5 years.

**Fig 3:**
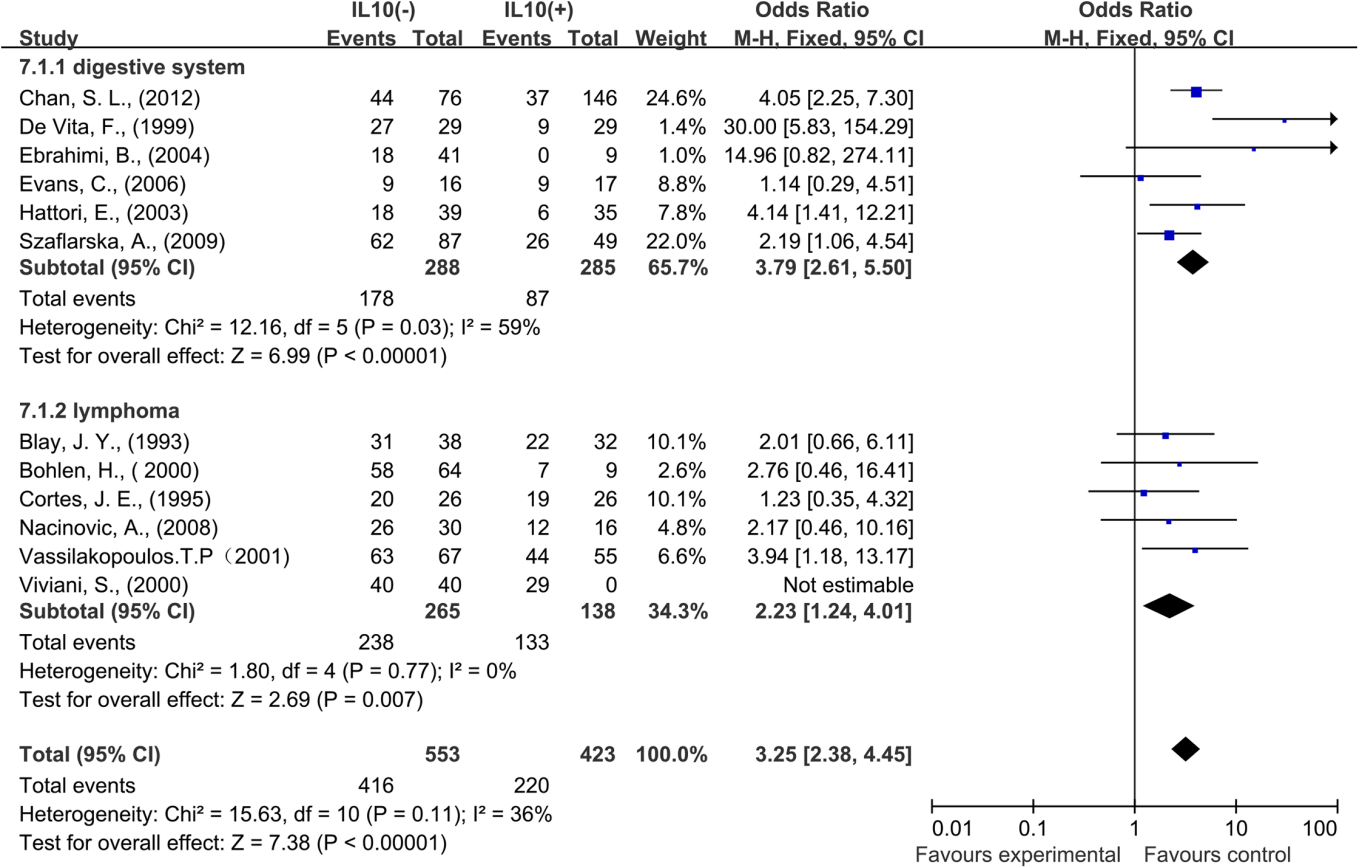
Forest plots describing analysis of the association between serous interleukin–10 (IL–10) expression and overall survival (OS) in digestive system cancer patients and lymphoma patients at 1 year.

A total of 16 studies reported data for OS at 1-year. Results showed that IL–10 overexpression was associated with worse 1-year OS in cancer patients (OR = 3.70, 95% CI = 2.81 to 4.87, P < 0.00001) (Fig 2). There was no significant heterogeneity among studies (OS: Cochran’s Q P = 0.04, I^2^ = 41%). In the stratified analysis by cancer types, IL–10 overexpression was associated with worse 3-year OS of digestive system cancer (OR = 3.79, 95% CI = 2.61 to 5.50, P < 0.0001) and lymphoma (OR = 2.23, 95% CI = 1.24 to 4.01, P < 0.0001) (Fig 3). As solid and hematological malignances were both included in our study, so we conducted subgroup meta-analysis to explore whether the results were consistent in different types of cancer.

**Fig 4:**
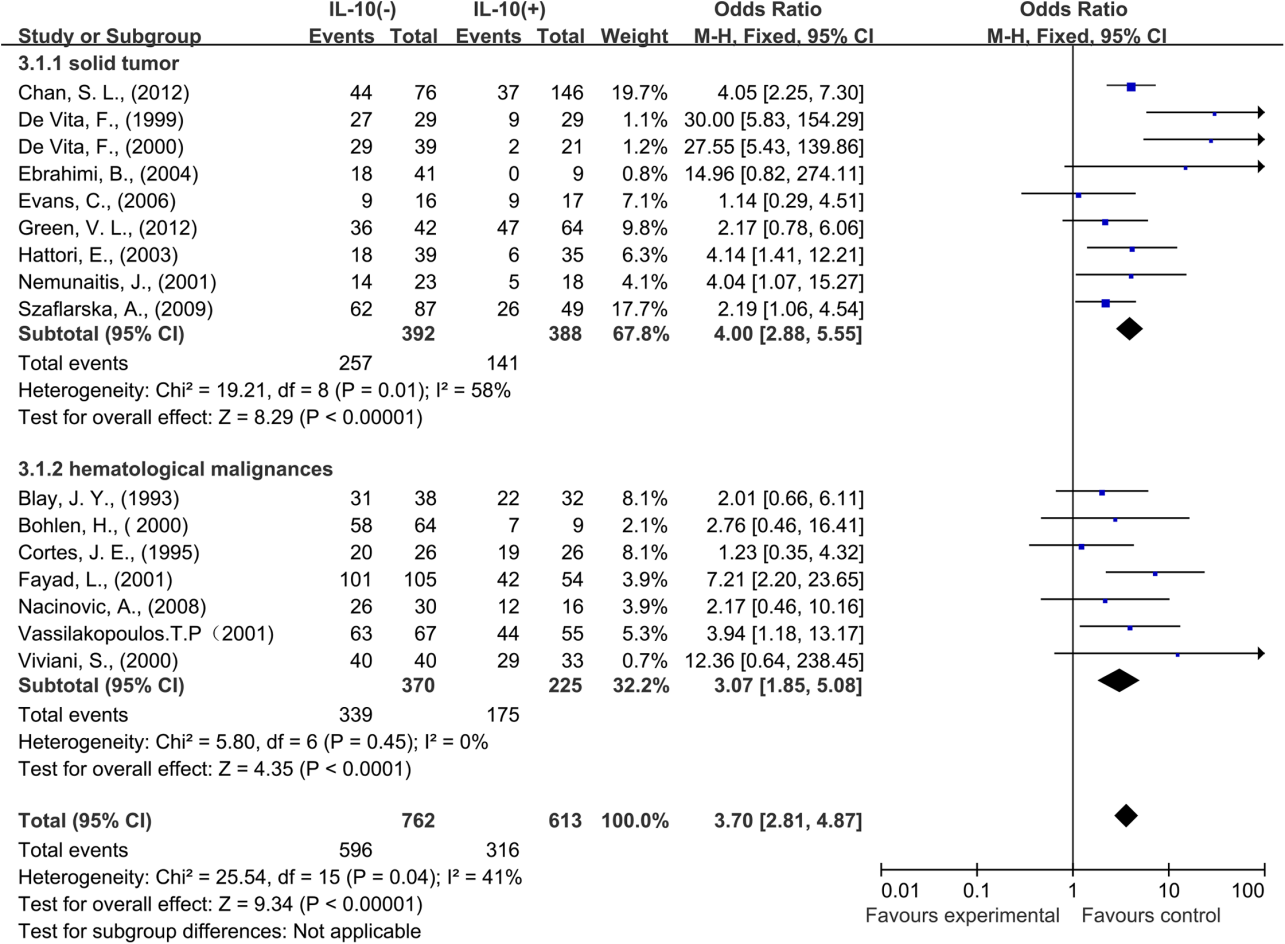
Forest plots describing subgroup analysis of the association between serous interleukin–10 (IL–10) expression and overall survival (OS) in solid tumor patients and hematological malignances patients at 1 year.

Nine studies provided 1-year OS for solid tumors, and seven studies for hematological malignances. In the stratified analysis by cancer types, IL–10 overexpression was associated with worse 1-year OS in solid tumors (OR = 4.00, 95% CI = 2.88 to 5.55, P < 0.0001). The similar result was also observed in hematological malignancies, high IL–10 level correlated with worse OS at 1 year (OR = 3.07, 95% CI = 1.85 to 5.08, P < 0.0001) (Fig 4).

A total of 15 studies reported data for OS at 3 years. IL–10 overexpression was also associated with worse 3-year OS in cancer patients (OR = 3.33, 95% CI = 2.53 to 4.39, P < 0.0001) (Fig 2). No significant heterogeneity was observed among studies (Cochran’s Q P = 0.12, I^2^ = 31%). Subgroup meta-analysis was also conducted to explore whether the results were consistent in different types of cancer.

**Fig 5:**
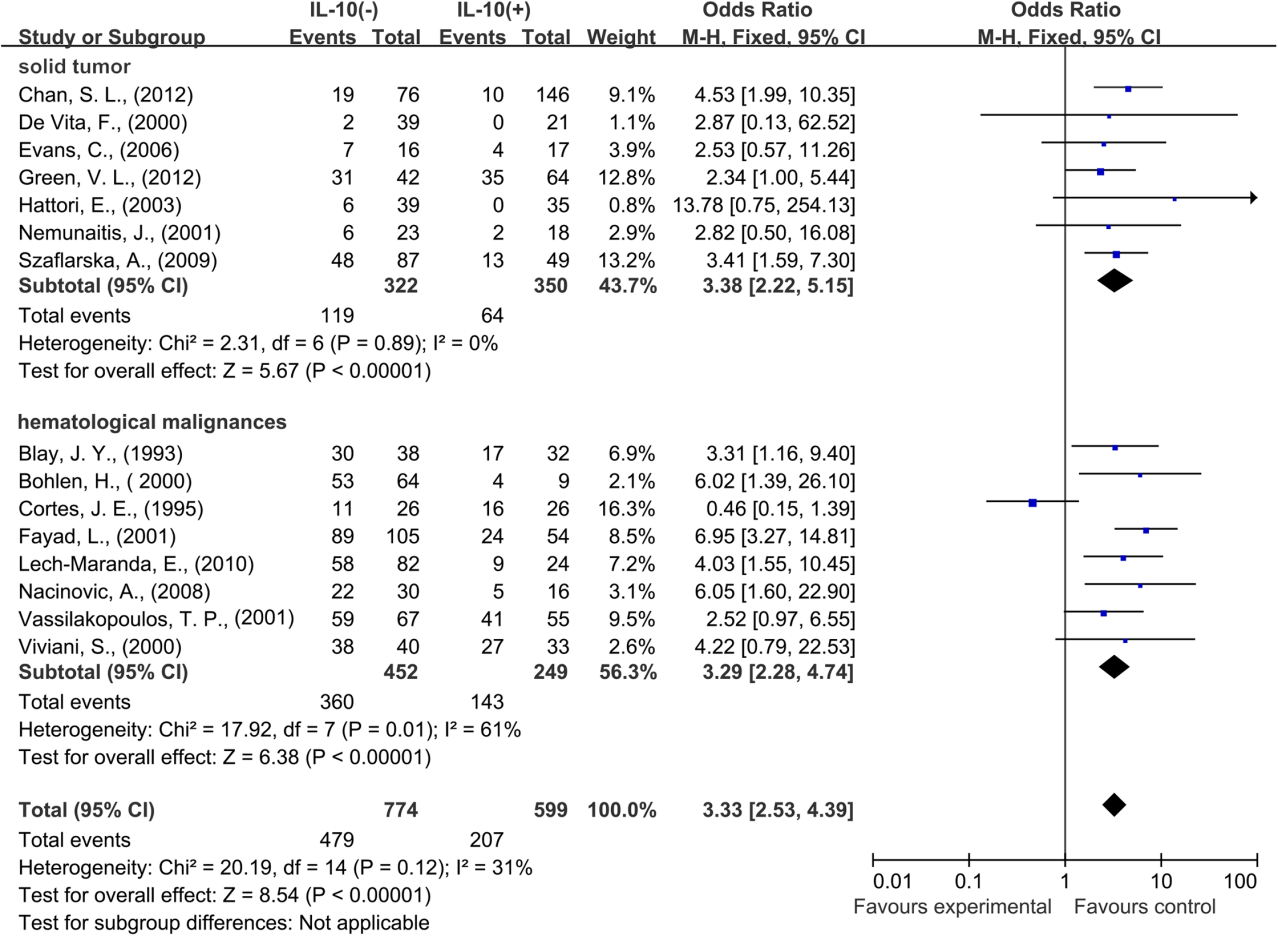
Forest plots describing subgroup analysis of the association between serous interleukin–10 (IL–10) expression and overall survival (OS) in solid tumor patients and hematological malignances patients at 3 years.

Seven studies provided 3-year OS for solid tumors, eight studies for hematological malignances. IL–10 overexpression was found to be associated with worse 3-year OS in both solid tumors (OR = 3.38, 95% CI = 2.22 to 5.15, P < 0.0001) and hematological malignances (OR = 3.29, 95% CI = 2.28 to 4.74, P < 0.0001) (Fig 5).

A total of 8 studies reported data for OS at 5-years. The result was similar to that of 1-year and 3-year, IL–10 overexpression was significantly associated with worse 5-year OS of cancer (OR = 2.80, 95% CI = 1.90 to 4.10, P < 0.0001) (Fig 2). There was no significant heterogeneity among studies (Cochran’s Q P = 0.35, I^2^ = 11%). Subgroup analysis showed that IL–10 overexpression was associated with worse 5-year OS in hematological malignancies (OR = 3.59, 95% CI = 2.26 to 5.72, P < 0.0001), however the amount of 5-year OS data in solid tumors was not enough for meta-analysis.

**Fig 6:**
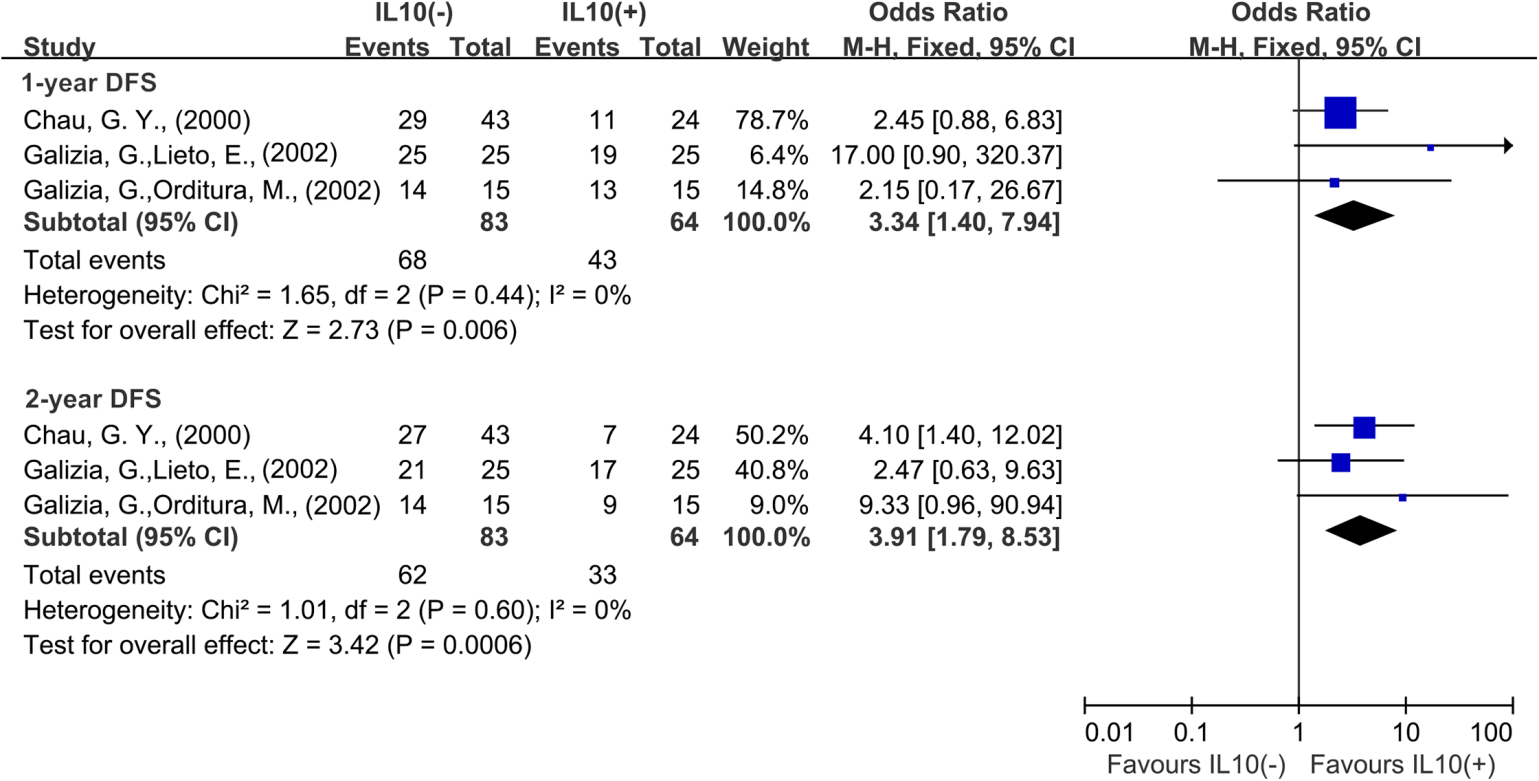
Forest plots describing odds ratios of the association between serous interleukin–10 (IL–10) expression and disease-free survival (DFS) at 1 year and 3 years.

A total of 3 studies reported data for DFS at 1 year and 2 years. Results showed that IL–10 overexpression was associated with worse 1-year DFS (OR = 3.34, 95% CI = 1.40 to 7.94, P = 0.0006) and worse 2-year DFS (OR = 3.91, 95% CI = 1.79 to 8.53, P = 0.0006) of cancer. There was no significant heterogeneity among studies (1 year: Cochran’s Q P = 0.44, I^2^ = 0%; 2 years: Cochran’s Q P = 0.60, I^2^ = 0%) (Fig 6).

### Sensitivity Analyses

Batch correction using the ComBat method had no important impact on the results for 1-year, 3-year or 5-year OS. The summary results were not significantly influenced when 3 studies which reported unsocial cut-off value were removed (OR = 4.05, 95% CI = 2.88 to 5.70; OR = 3.36, 95% CI = 2.42 to 4.66; OR = 3.48, 95% CI = 2.12 to 5.69, respectively). No obvious heterogeneity was observed after exclusion of these studies (Cochran’s Q P = 0.02, I^2^ = 49%, Cochran’s Q P = 0.06, I^2^ = 42%; Cochran’s Q P = 0.89, I^2^ = 0%, respectively).

## Discussion

IL–10 shows its bidirectional functions both in pro-tumor and anti-tumor effect. In previous cancer studies, high level of IL–10 was reported to correlate with poor clinical outcome [29], while some others suggested IL–10 as a beneficial factor in cancer prognosis [32]. Here we meta-analyzed the published data about the expression of IL–10 in serum of 1788 cancer patients from 21 published studies and their association with survival for studies that evaluated IL–10 by ELISA.

IL–10 was generally known as an immunosuppressive cytokine which mainly promoted the proliferation and metastasis of tumor cells [20]. However, IL–10 was newly found to active anti-tumor immunity in tumor microenvironment [36]. Our results showed that high level of IL–10 in serum was associated with worse 1, 3, and 5-year OS and worse 1-year DFS for 21 studies analyzed totally, suggesting IL–10 is a potential biomarker for the prognosis evaluation and IL-10-targeted therapy. We believe that our study provides significative statistical evidence to declare the important prognostic value of IL–10 as a tumor promoter in cancer patients for the first time.

Actually, IL–10 has been explored as a novel therapeutic target for a long time. Significant restrain of colon and breast tumors in mice was induced by delivery of anti-IL-10-receptor antibody in combination with CpG and CCL16, which supported the effectiveness of anti-IL–10 therapy [35]. In contrast, a recent study reported that recombined IL–10 induced tumor rejections by specifically targeting the tumor-infiltrating memory CD8+ T cells to activate the anti-tumor immune response [36]. Therefore, applying IL–10 antagonist or recombinant IL–10 is still a matter of some debate. Our findings provide statistical evidence for this argument. High level of serum IL–10 showed poor prognosis of cancer patients, suggesting IL–10 antagonist may be a novel therapeutic technique for clinical treatment of cancer patients.

Subgroup analysis of solid tumors and hematological malignancies was conducted to identify the effect of IL–10 on different types of cancer. IL–10 showed a close correlation with poor prognosis of both solid tumors and hematological malignancies. Interestingly, patients with solid tumors showed mild higher ORs at 1-year and 3-year than patients with hematological malignancies. Among specific cancer types, both digestive system cancer and lymphoma linked with a poor prognosis for patients who expressed high serum IL–10. This finding suggests that IL–10 plays a crucial role in tumor progression both in solid tumors and hematological malignancies, probably by inducing systemic immunosuppression.

Several important implications were put forward in these analyses. First, we show that high level of serum IL–10 has a tight correlation with poor prognosis in cancer patients, which suggests that IL–10 is a promising biomarker for the evaluation of disease progression and survival time. Second, it provides statistical evidence to support that IL–10 antagonist in anticancer target therapy could have a better response than recombinant human IL–10. Finally, this analysis emphasizes the discrepant application of IL-10-targeted drugs toward solid tumors and hematological malignancies. Following these discoveries, further clinic trials on IL–10 antagonist is imperatively needed to detect the clinical response in cancer patients.

Several limitations should be noted from this analysis. It is hard to eliminate the publication bias in a literature-based analysis. The studies reporting negative results may have few chances to be published. Besides, separate analysis for every types of cancer was not allowed in this study because some types of cancer did not have enough data. The solid/hematological-tumor differentiation may increase the heterogeneity in each subgroup.

In conclusion, our analyses show that high level of serum IL–10 is associated with worse clinical outcome of cancer patients, which indicates that IL–10 might be a potential biomarker for prognostic prediction and targeted treatment in human cancer. Additionally, our findings present statistical evidence for the clinical application of IL–10 antagonists in cancer therapy.

## Supporting Information

S1 Table Characteristics of the included data. (DOCX)

## Abbreviations

IL–10: interleukin–10
OS: overall survival
DFS: disease-free survival
OR: odds ratios
Cl: confidence interval
ELISA: Enzyme-Linked Immunosorbent Assay
