# Supplementary material for: Serum IL-10 Predicts Worse Outcome in Cancer Patients: A Meta-Analysis
Source: PLoS One. 2015 Oct 6;10(10):e0139598. doi: 10.1371/journal.pone.0139598 (PMC4595202; doi:10.1371/journal.pone.0139598)
Supplement: S1 Table — (DOCX) [file pone.0139598.s001.docx]

| References | Type of cancer | patientNO. | Age(range) | Male/female | stage | Cut-off | Follow up months (range) | IL-10(-/+) NO. | 1-y OS(-/+)% | 3-y OS(-/+)% | 5-y OS(-/+)% | Quality score (NOS) |
| --- | --- | --- | --- | --- | --- | --- | --- | --- | --- | --- | --- | --- |
| Alhamarneh, O., et al.(2011)[38] | head and neck squamous cell carcinoma | 107 | 64（26-90） | 85/22 | I-IV | 0.2 pg/ml | 15(1–36) | 66/41 | 90/78 | 74.4/52.8 | NR | 8 |
| Blay, J. Y., et al.(1993) [39] | Non-Hodgkin’s Lymphoma | 70 | 46 (3-89) | NR | I-IV | 100 pg/mL | NR | 32/38 | 81.4/67.9 | 78/53.6 | 78/49.3 | 8 |
| Chan, S. L., et al. (2012) [40] | hepatocellular carcinoma | 222 | 59.9±12.3 | 198/24 | I-IV | 1.00 pg/mL | 31（0-48） | 146/76 | 57.7/25.6 | 25.6/7 | NR | 8 |
| Cortes, J. E., et al. (1995) [41] | Diffuse Large Cell Lymphoma | 52 | 56(24-78) | 32/20 | I-IV | 8 pg/ml | 26(12-44) | 26/26 | 75.5/73.6 | 43.7/61.4 | NR | 7 |
| De Vita, F., et al. (1999) [42] | Gastrointestinal Malignancies | 58 | <60:50%. >60:50% | 39/19 | III-IV | 18 pg/mL | 12.9±6.4 | 29/29 | 92.4/32.1 | NR | NR | 7 |
| De Vita, F., et al. (2000) [43] | Non-small Cell Lung Cancer | 60 | <60:46.7 >60:53.3% | 49/11 | III-IV | 19.6 pg/mL | 12.8±7.8 | 21/39 | 74.3/9.6 | 5.1/0 | NR | 8 |
| Ebrahimi, B., et al. (2004) [44] | Pancreatic Carcinoma | 50 | 65(43-79) | 30/20 | NR | 9.8 pg/ml | 10(0-22) | 9/41 | 44/0 | NR | NR | 7 |
| Evans, C., et al. (2006) [45] | colorectal cancer | 33 | 76.5 | 22/11 | NR | NR | 89 | 17/16 | 59/53.1 | 41.2/23.6 | 37.5/17.6 | 7 |
| Fayad, L., et al. (2001) [46] | chronic lymphocytic leukemia | 159 | 60(21-82) | NR | I-IV | 10 pg/ml | 30(1-40) | 54/105 | 96.4/78.3 | 85/45 | NR | 8 |
| Green, V. L., et al. (2012) [47] | head and neck squamous cell carcinoma | 106 | 64（26-90） | 84/22 | I-IV | 0.2 pg/ml | 33(1-67) | 64/42 | 85.3/73.3 | 74.4/54.6 | 59.1/54.6 | 8 |
| Hattori, E., et al. (2003) [48] | hepatocellular carcinoma | 74 | 65（41–88） | 54/20 | I-IV | 10 pg/ml | NR | 35/39 | 45/16.8 | 15.5/0 | NR | 8 |
| Lech-Maranda, E., et al. (2010) [49] | Diffuse Large B-Cell Lymphoma | 106 | <60:46.2%>60:53.8% | 50/56 | I-IV | 5 pg/ml | NR | 24/82 | NR | 70.7/36 | NR | 8 |
| Lech-Maranda, E., et al. (2012) [50] | chronic lymphocytic leukemia | 160 | <60:32.5%>60:67.5% | 86/74 | I-IV | 17.8 pg/ml | 48(1.2-200) | 82/78 | NR | NR | 92.1/80.52 | 8 |
| Nacinovic, A., et al. (2008) [51] | diffuse large B-cell lymphoma | 46 | 58 (17-82) | 25/21 | I-IV | 20.2 pg/ml | 50(1-69) | 16/30 | 85.8/74.9 | 75/31 | 71/25 | 7 |
| Nemunaitis, J., et al. (2001) [52] | Melanoma | 41 | NR | NR | advance | 10 pg/ml | NR | 18/23 | 60.5/28.1 | 27.3/11.4 | NR | 7 |
| Szaflarska, A., et al. (2009) [53] | Gastric Cancer | 136 | 61.5±11.9 | 84/52 | I-IV | 10 pg/ml | 82.6 | 49/87 | 71.6/52.9 | 55.4/26.2 | NR | 7 |
| Vassilakopoulos, T. P., et al. (2001) [54] | Hodgkin’s lymphoma | 122 | 31.5(15-76) | 74/48 | I-IV | 10 pg/mL | 27(6-136) | 55/67 | 93.4/79.7 | 88.3/75 | 85.4/66.5 | 8 |
| Viviani, S., et al. (2000) [55] | Hodgkin's disease | 73 | 27(17-61) | 44/29 | I-IV | 6 pg/ml | 7.3y(1-8y) | 33/40 | 100/87.8 | 95.4/82 | 95.4/79 | 8 |
| Chau, G. Y., et al. (2000) [56] | Hepatocellular Carcinoma | 67 | 63.4±1.5 | 60/7 | NR | 12 pg/mL | NR | 21/46 | 66.3/46.6 |  |  | 8 |
| Galizia, G., Orditura,M., et al. (2002) [57] | Colon Cancer | 30 | 65.4±10.5（30-83） | NR | NR | 15 pg/mL | 22.2±6.6（5.2-26） | 15/15 | 93.3/86.2 |  |  | 7 |
| Galizia, G., Lieto,E., et al. (2002) [58] | Colon Cancer | 50 | 65.4±10.5 (37-83） | 34/16 | NR | 14 pg/mL | 15.5±6.7 (0.3-26) | 25/25 | 100/75.5 |  |  | 7 |

S1 Table:

Characteristics of the included data.
